# Quality Evaluation of Selected Organic Coatings Used on Roofing Sheets

**DOI:** 10.3390/ma15041310

**Published:** 2022-02-10

**Authors:** Krzysztof Przystupa

**Affiliations:** Department of Automation, Lublin University of Technology, Nadbystrzycka 36, 20-618 Lublin, Poland; k.przystupa@pollub.pl

**Keywords:** quality, organic coatings, roofing sheet metal, scratch test σ

## Abstract

This paper discusses the aspects of quality evaluation of organic coatings on roofing sheet surfaces. Scratch resistance was defined as a quality property of an industrial product. The research was comparative and exploratory in terms of the method applied and research results. The study followed a quantitative and qualitative approach in which evaluation is based on several parameters, such as the mechanical behavior of a coating in a scratch test, profile and depth of remained damage, and microscopic evaluation of damage mechanisms. The study parametrically describes coating damage and destruction mechanisms. It has been shown that the resistance of the coatings is not identical, and the research results confirm that the applied approach is relevant to evaluate qualitative features of roofing sheet metal coatings.

## 1. Introduction

The word quality derives from the Greek word qualitas and has been incorporated into many languages. Professor Kilinski’s fundamental study [1] defines quality in many ways. The quality of technical objects is defined as follows: “The quality of an object is the set of its inherent properties that define the degree to which an object is able to satisfy user’s requirements” [2]. At the current stage of social development, industrial products are common in use, but the management of limited resources is respected so there is social pressure to enhance the use value of industrial products. Quality can be evaluated if there are evaluation measures that are typical for a given product [3,4]. Evaluation measures can be physical quantities, e.g., temperature, which means so-called measurable quality characteristics that can be sometimes descriptive and non-measurable, e.g., taste. Measurable quality characteristics are, however, applied to paint coatings, and it is very rare to evaluate such coatings by electronic measurements such as color, which is an example of a non-measurable characteristic. Basic types of measurements are technical, economic, and utilitarian [5,6]. Technical measures are obviously particularly important for researchers in engineering and technology. These measures relate to both entire technical objects and their parts. Active parts, basic and spare, that condition direct operation are fundamental, but passive parts that have an impact on convenient use, quality, and aesthetics are also important [7]. Organic coatings that coat the surfaces of metal thin-walled components have an impact on characteristics of both groups of parts [8]. This means that the characteristics of such coatings are a key criterion for the quality evaluation of technical objects. Important aspects for quality evaluation are reliability and durability related to resistance to damage and time to retain functional properties [9]. Typical kinds of damage include those perceived as a physical phenomenon [10]. They can be related to the destruction of an element or its surface (e.g., fracture, chipping, wear, corrosion, and aging) and not related to destruction (e.g., clogged fuel supply lines, loosened connections, dirty contacts, etc.) [8,11]. Mechanisms of damage in operation of technical objects may be different, but they are always related to the action of forcing factors. Surface scratching may occur in production, assembly, transport and operation in expected working conditions [12]. Warszyński [11] classifies this type of damage as normal wear and tear. It is known, however, that such a type of damage, although unacceptable, can also occur in production. The three main aspects in scratch resistance testing are aesthetics, structural integrity, and durability. Aesthetic aspects are important for many products, e.g., signboards or cell phones. Any visible scratches on such products reduce their quality, although their functionality remains definitely unchanged. Durability is impacted by surface scratches that lead to damage spreading below a surface, e.g., damaged polymer coatings of steel pipes cannot prevent the corrosion of metal parts below. Structural integrity is, for example, related to damage to packaging [13]. Scratch resistance and adhesion to the substrate are actually one of the main measures of resistance for organic roofing sheet metal coatings. Expert engineers classify scratches as the main damage to metal roofing sheets and describe in practical terms causes of scratch damage [14] because scratches are one of the causes of damage. Researchers have attempted, although with mixed success, to relate scratch resistance to other mechanical properties, such as stiffness, hardness, and viscoelasticity [15]. Many of these methods, however, require special samples that, e.g., are of different sizes than a real product, and the best way to describe properties of a coating is to apply the coating to a real substrate in line with an industrial technological regime. Currently, there are industrial methods for evaluating scratch resistance, and one of the normative methods is the Clemen’s method, as specified in PN-EN ISO 1518:2000 [16]. This method enables us to evaluate coating quality by a scratch criterion, and measurements are given in grams. A scientific approach to describe the scratch resistance of coatings is a test that records scratch depth and visualizes this scratch [17]. More sophisticated approaches involve applying a gradually increasing force and recording quantities used to describe a scratching process, e.g., variation in the frictional force, depth of residual scratching (Rd), and under load (Pd) [18]. There is also an approach with a synergistic evaluation of variation in the frictional force and acoustic emission signal as well as a qualitative evaluation of coating damage. Such a method has just been applied here. An instrumental method gives more accurate results, so this study follows a comprehensive evaluation method that combines quantitative and qualitative approaches. This method is also used by specialized measuring equipment and shows a higher accuracy and scientific level of analysis—beyond the accepted standards and industrial methods. It is known that coatings of roofing sheets are varied. They differ, among others, in surface topography. This state of affairs makes it difficult to compare functional features. The aim of the study is to assess the quality of various roofing sheets and the possibility of using instrumental methods to assess their properties. The tested products are sheets of metal with a different structure, different mechanical properties, and a different surface topography, popular in Poland.

## 2. Materials and Methods

The tested samples were taken from real industrial products. The material was coatings applied on sheet metal surfaces marked by their manufacturer, ArcelorMittal Poland (Dąbrowa Górnicza, Poland), as ZA200 (marked here as “black”—MAT 7024, steel and coating parameters are given in [19]) and S220GD (marked here as “white”—RAL 9010, steel and coating parameters are given in [20]). The sheets are covered with multi-layer polyester and polyurethane coatings. Polish roofing sellers claim that the products tested in the article are of excellent quality, highly durable and usable. The sellers do not indicate any functional or technical differences.

The scratch resistance and adhesion of the coatings to a metal substrate were tested with a Micro Combi Tester (MCT) by Anton Paar. A Rockwell indenter as a diamond cone of a tip radius of 100 µm roundness was loaded incrementally at a rate of 2 N/min in the axial direction from a normal force (Fn) of 0.03 N to a force of 10 N. The scratch test was performed for a 10 mm length at a speed of 2 mm/min. The following quantities were recorded in the test at 30 Hz: friction coefficient (μ), friction force (Ft), indenter penetration depth (Pd) and depth remained after scratching (Rd). The surface profile was identified by pre- and post-scans.

The surface profiles of the samples were tested using a Bruker ContourGT-I 3D Optical Microscope (Bruker, Fällanden, Switerzland) capable of scanning surfaces up to 10 mm. Its vertical resolution is <0.01 nm, its horizontal resolution is up to 0.01 µm, and its scanning speed is up to 73 m/s. In this study, a surface of 1 mm × 1 mm was scanned in line with PN-EN ISO 4287:1999 [21].

The nanoindentation tests identified the mechanical properties of the protective coatings. The samples were prepared for the tests so that it was possible to measure the metal sheet in its cross-section. For this purpose, the samples were mounted in a thermosetting phenolic polymer resin and metallographed in an ATM Opal hot mounting press (ATM Qness GmbH, Mammelzen, Germany). The microsections were polished with a Saphir 550 Opal automatic laboratory grinder and polisher (ATM Qness GmbH, Mammelzen, Germany) using MD Piano abrasive discs with grit sizes of 120, 240, 500, and 1200. The samples were water cooled and then polished with polishing cloths and 3 μm particle size polishing diamond suspension. The prepared microsections were examined with an UNHT nanoindentation hardness tester (Anton Paar GmbH, Graz, Österreich). The recorded parameters included the hardness and modulus of elasticity of the coatings.

## 3. Results and Discussion

**Figure 1:**
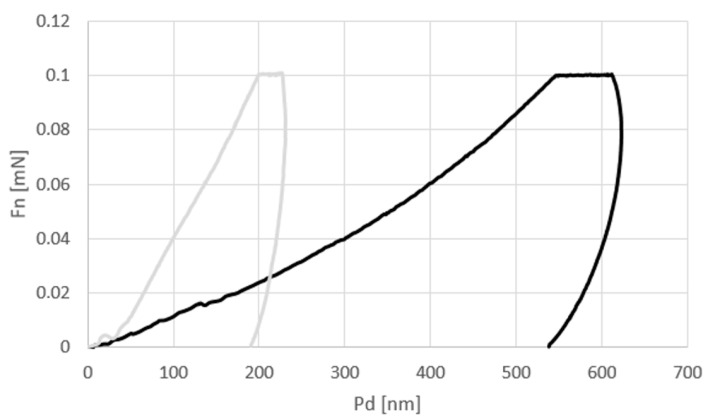
Average Fn vs. Pd from the indentation hardness tests.

Figure 1 shows the plot of the average normal force (Fn) vs. penetration depth of the indenter into the coating (Pd). The shapes and ranges of the curves are very different. The range of Pd is much larger for the “black” coating and is approximately five times more than for the “white” one.

The nanoindentation hardness test specified the mechanical and elastic parameters of the tested surfaces. The indentation hardness *H_IT_* (1) was specified from the correlation between *P*_max_, i.e., the highest normal force loading the indenter and the indenter contact area under the maximum load *A* and in line with the following Equation [22]:

(1) $$H_{IT}=\frac{P_{\max}}{A}$$

The converted Vickers hardness was calculated from Equation (2) [22]:

(2) $$HV_{IT}\approx \frac{H_{IT}}{10.58}$$

The surface elastic modulus *E_IT_* was calculated from the stiffness specified from Equation (3) [22]:

(3) $$S=\frac{dP}{dh}=\beta \cdot \frac{2}{\sqrt{\pi }}\cdot E^{*}\cdot \sqrt{A}$$

The relation $\frac{dP}{dh}$ was determined from the force vs. indenter displacement plot (Figure 1). In Equation (3), the parameter β is taken equal to 1 for any symmetric indenter. For a Vickers indenter, the corrected value of parameter *β* = 1.0055 [22]. The quantity *A* is a function of depth *h_c_* and is calculated from Equation (4) according to [23]:

(4) $$A=F\left(h_{c}\right)=24.54h_{c}^{2}+C_{1}h_{c}^{1}+C_{2}h_{c}^{1/2}+C_{3}h_{c}^{1/4}+C_{4}h_{c}^{1/8}+\ldots +C_{n}h_{c}^{1/2n}$$

In Equation (4), the calculation is based on a constant *C_n_* that expresses the indenter geometry. The method for determining the constant *C_n_* is described in [24]. In the stiffness calculations, a modulus of the tested surface layer, *E*, is defined by Equation (5):

(5) $$\frac{1}{E^{*}}=\frac{1-\nu^{2}}{E}+\frac{1-\nu_{i}^{2}}{E_{i}}$$

The descriptive statistics of the parameters are presented in Table 1.

The value E* in Equation (5) is the reduced elastic modulus and *ν* is the Poisson’s number of the sample. The value E* reflects elastic deformations both in the tested sample and the indenter, which is discussed in more detail in [22]. The symbols of *Ei* and *νi* refer to the indenter. Equation (5) is a general correlation that can be applied to any symmetric indenter and is not limited to a particular geometry, such as a cone or pyramid. Equation (5) was originally introduced for elastic contact but has turned out to also be correct for elastic–plastic contact [22].

The parameter *E_IT_* was calculated from Equation (6), which uses the stiffness *S* estimated from Equation (3) and the penetration path of the indenter corresponding to the elastic deformation of the tested surface *hc*:

(6) $$E_{IT}=\frac{\sqrt{\pi }S}{2\beta \sqrt{A\cdot hc}}$$

The nanomechanical properties of the two coatings differ significantly. The indentation hardness of the “white” coating is almost 22 times higher than that of the “black” coating. The elastic moduli of the two coatings differ even more. The mean indentation elasticity modulus of the “white” coating is approx. 32 times higher than the elasticity modulus of the “black” coating. The damage and deformation of coatings depend largely on the mechanical properties of coatings [25]. The very large variation in the mechanical properties of coatings was attempted to relate to the results of the surface profile and scratch tests.

**Figure 2:**
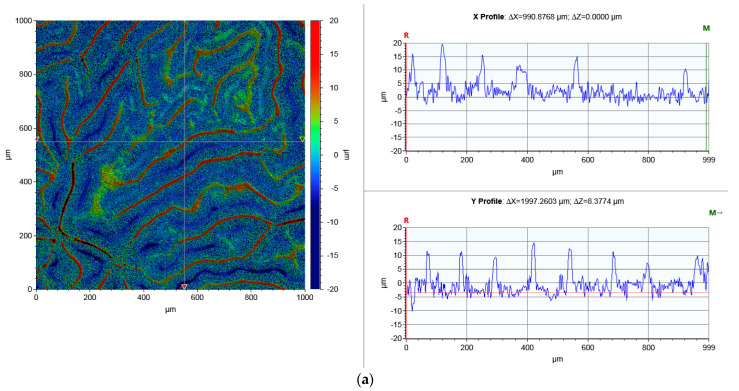

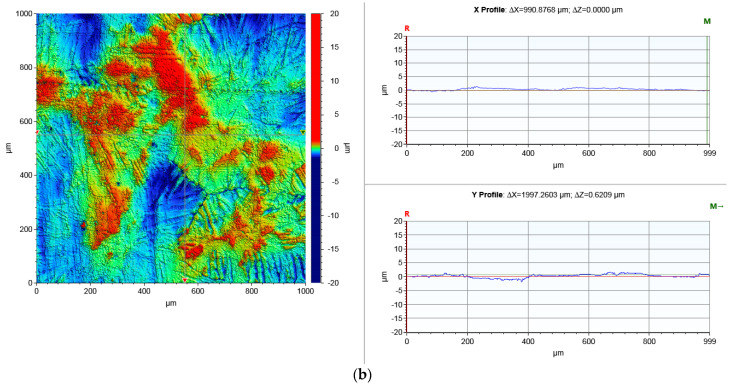
Selected profilogram of the coating surfaces of the “black” (**a**) and “white” (**b**) coatings. Coatings surface topography and selected profilograms from the X and Y directions of the coordinate system axis (marked with lines on the colored topographic chart).

Figure 2 present the findings for the coating surface profile tests. The specified parameters include the arithmetic mean of the profile deviations from the mean line *Ra* and the total height of the roughness profile *Rt* (Table 2).

The “black” coating showed a higher roughness. The *Ra* parameter of the “black” coating surface was about six times higher than that of the “white” one. A similar correlation was found for the total height of the roughness profile. A characteristic wave-like shape formed on the “black” coating surface (Figure 2). Moreover, the profilograms (Figure 2) confirm that the “black” coating’s roughness profile was more expanded than that of the “white” coating. Significant differences in the measured amplitude parameters of the roughness profile can have an impact on performance parameters. It has been reported [26] that the *Ra* parameter is important for contact stiffness, thermal and electrical conductivity, and friction and wear and tear. The roughness profile of the “black” metal sheet indicates that waviness, apart from height parameters, may also be important. According to [27], the total height of the roughness profile (*Rt*) affects surface reflectivity and mechanical tightness. The two roughness height parameters that were measured are also related to bearing capacity, contact stresses, and fracture toughness [28]. The roughness parameters of the two coatings are different and so their resistance should also be assumed to be different.

**Figure 3:**
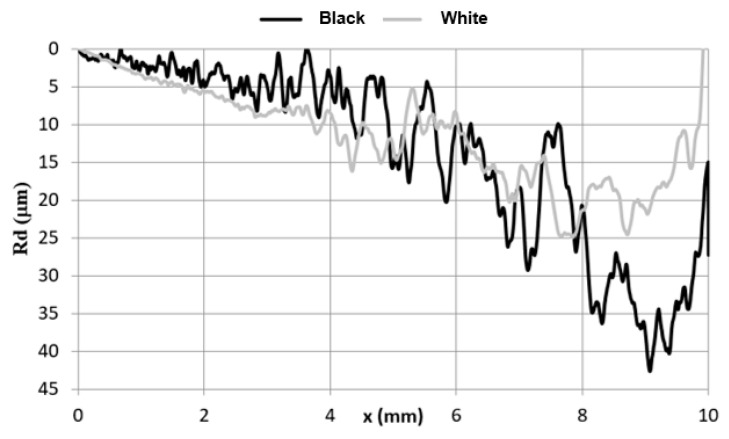
Average permanent crack depth (Rd) in the roof sheet coatings.

**Figure 4:**
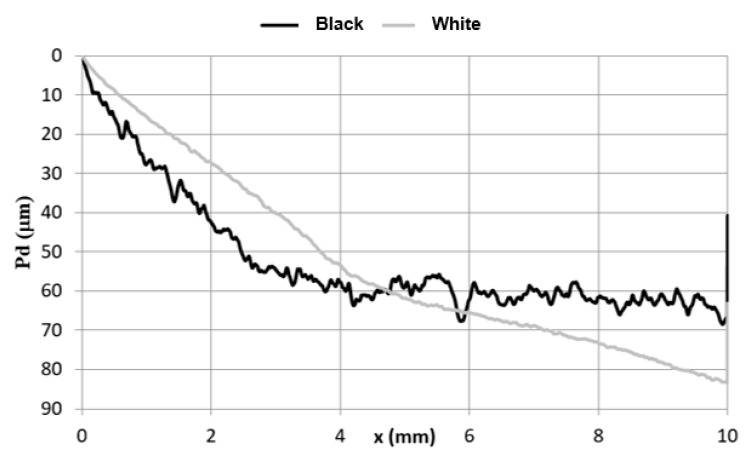
Average indenter penetration depth (Pd) in the roof sheet coatings.

**Figure 5:**
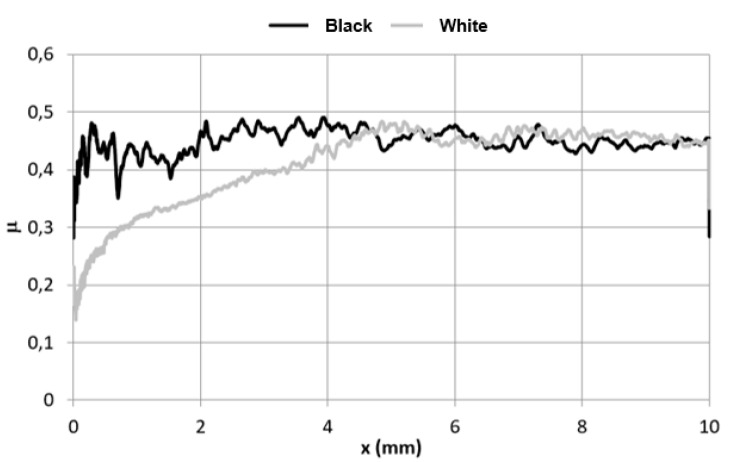
Average friction coefficient (µ) courses in the scratch test.

It is known that the resistance of coatings does not merely depend on the geometrical properties of a roughness profile but also on the structure and properties of coating materials. Generally speaking, the wear and tear of polymers depends on the hardness of materials in a friction node, and operating wear and tear is caused by micro-cutting, scratching, and furrowing [19]. It is not obvious why a given material is resistant to scratching and surface damage. Only an objective methodology based on the principles of materials engineering allows for an unambiguous evaluation [13]. Most polymers are, unfortunately, prone to scratch damage [13], and it is important to understand the extent to which they are. The same also applies to polymer coatings on a metal substrate. Given these roughness measurements and a suitable method, damage to the coatings was studied. Mechanical analysis of a scratch test, especially for polymers in which a large elastic portion accompanies plastic deformation, is very complex [29]. The parameters that describe the surface damage, or cracks in the tested materials, are depicted in the graphs (Figure 3, Figure 4 and Figure 5).

The average curves that correspond to the permanent scratch depth (Rd) are summarized in the graph (Figure 3). The shapes of the Rd curves of the two coatings are different. The curves for the “black” coating are very irregular, and the inter-peak values are as high as 20 µm. Such a value is justified by a maximum height of the profile, and the Rt parameter is higher than 45 µm (Table 1). This is probably related to the technological roughness of this coating and its thickness, but not only, as indicated, for example, by the graph (Figure 1) and the course of the deadhesion of the coating (Figure 4). Such a mechanism of destruction probably results from a type of coating material. It has been found that a material pile-up forms in some polymers, and such a material accumulates in front of an indenter and rotates around it [30]. A similar deformation in front of and under an indenter was already observed when some metals were scratched [30]. This phenomenon is called a frontal pile-up [30,31]. It has been found [30] that lifting out an indenter due to a material accumulated under it depends on the so-called rheological coefficient. Figure 4 clearly depicts that the indenter does not reach the metallic substrate in every case and is lifted out. This seems to be a synergistic effect of the technological unevenness (Table 1) and the material accumulated under the indenter. In the final stage of the test, a deeper scratch in the “black” coating was observed, which means that this material is more prone to scratching. This fact was verified by the under-load behavior of the coating. Indenter penetration depth into the tested coatings under the test load is plotted in Figure 4. The curves for both tested coatings do not overlap. At the first stage of crack formation, the indenter penetrates the material deeper in the “black” coating, and then the curves collapse probably because the indenter approaches and contacts with the metal substrate.

Figure 5 shows the average courses of the friction coefficient (μ), and the curves do not overlap. The friction force corresponding to the resistance of the material to scratching is higher for the “black” coating in the friction path up to 4 mm. The friction coefficient, μ, is also clearly non-linear, which is probably related to the mechanism of destruction described later. It has been shown [32] that as the contact force increases, the friction coefficient increases to a small extent. The tests of the roofing sheet metal coatings show such a dependence only at the initial stage of the scratch test for the “white” coating. At the next stage, until the end of the test, the values of the friction coefficient were quasi-constant. The negligible cyclic fluctuations of the friction coefficient are manifested by a sinusoidal (or triangular) course of the friction coefficient. Such a shape is probably related to the cyclic strengthening and cohesive destruction of the coating as well as the above-described synergistic impact of technological unevenness and frontal pile-up.

**Figure 6:**
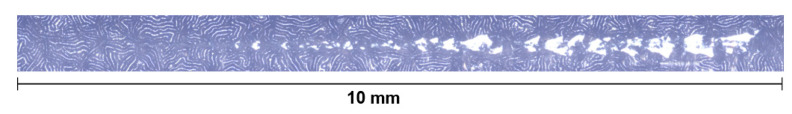
Optical microscope image of the scratched “black” protective coating (Olympus, objective, with 20× magnification, aperture of 0.4).

**Figure 7:**
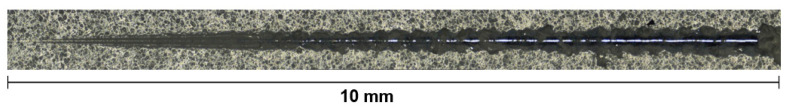
Optical microscope image of the scratched “white” protective coating (Olympus, objective, with 20× magnification, aperture of 0.4).

Figure 6 and Figure 7 show the optical microscope images of scratches on the coating. It is easy to notice the damage caused by micro-scratching. The bright fields in the microscope images correspond to the surface zones where the coating is deadhesion. These are so-called adhesion defects. Cohesive damage such as micro-cracking and chipping is also clear in the “black” coating. Wedging-type adhesive damage is noticeable on the “black” -coated surfaces. Wedging is caused by a delaminated area wedged against the coating being separated [33], and visible as damage of relatively regular shapes, e.g., ellipses, arcs, etc. This probably relates to a frontal pile-up phenomenon.

**Figure 8:**
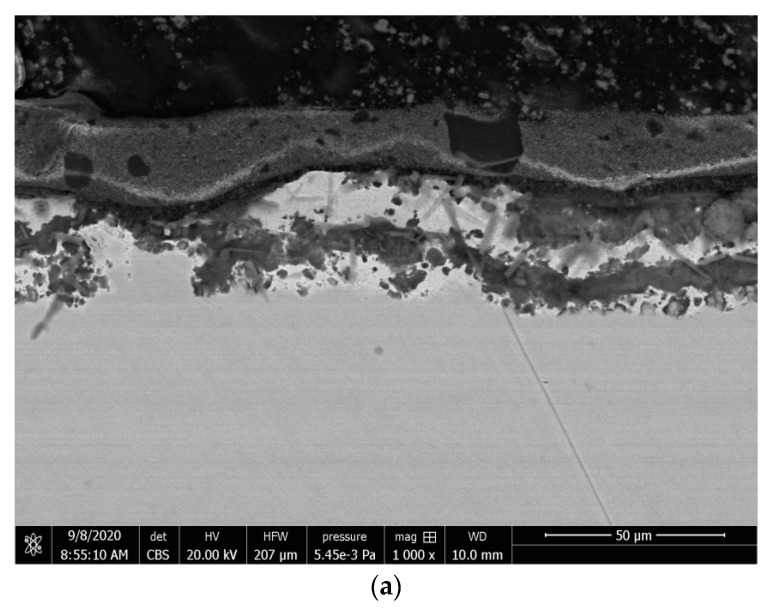

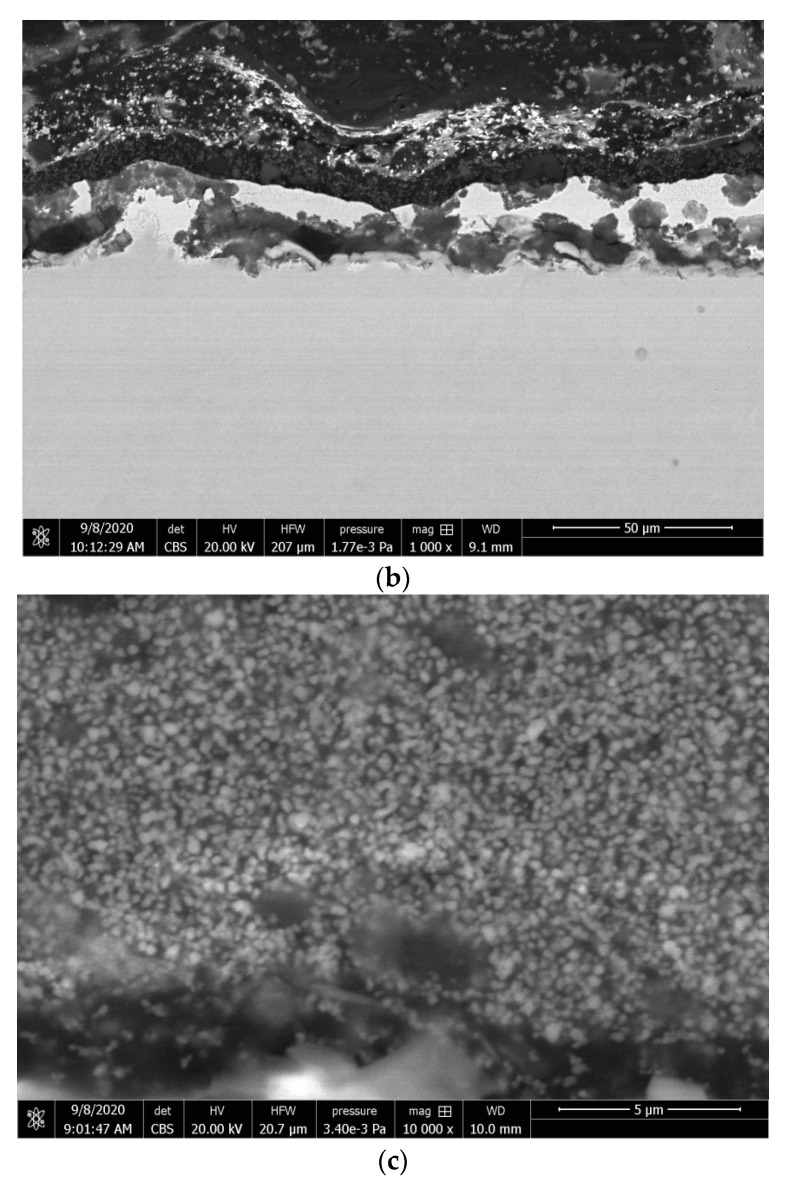
SEM images of the investigated coatings: (**a**) cross-section of the “white” coating (1000×); (**b**) cross-section of the “black” coating (1000×); (**c**) cross-section of the “white” coating (10,000×).

The above-described way the coating operates under concentrated load probably results from its structure. The SEM images of the cross-sections of the investigated coatings in Figure 8 show that the coatings differ structurally. The “white” coating has two main layers (Figure 8a,c), and its layer at the contact with the metal substrate and the metal substrate seem to form a hybrid layer. The “black” coating (Figure 8b) has three main layers. The boundary between the metal substrate and the contacting layer of the “black” coating is much more distinct than that of the “white” coating. It is likely that the adhesion, or the surface bonding of layers of two different bodies that are in contact with each other due to intermolecular attraction [34], of the two coatings investigated will be different. Adhesion is caused by many mechanisms, i.e., physical adsorption, chemical adsorption, diffusion, energy and electrostatic phenomena [35,36]. The coating is mechanically connected to the substrate by anchoring on macroinectroins as well as on micro- and submicroins of the surface. Physical–chemical interactions, i.e., physical and chemical adsorption, are, of course, also possible. Diffusion can also occur, which is typical of a hybrid layer. Diffusion is the phenomenon of joining particles of two materials that are in direct contact. Besides adhesion, the phenomenon of cohesion, or the mutual attraction of particles due to cohesive (intermolecular) forces [37], is fundamental for the connections between the coating and the substrate. Moreover, the thickness of the “black” coating is more irregular than that of the “white” one. In fact, there are no industrial standards that could specify minimum and maximum thicknesses of protective coatings [35,36], but it is known that coating thickness results in usable quality, e.g., insufficient thickness may lead to corrosion and excessive thickness may cause problems such as coating settling, flaking and cracking [35,36].

The performance properties tested can also be impacted by the phase structure. The outer layer of the “white” coating is composite. Its matrix is reinforced with a powder filler, and inorganic particles are probably visible. The share of the filler is very high and seems to be much higher than in the outer layer of the “black” coating. It has been shown that the share of a powder filler in organic–inorganic composites significantly affects performance [38]. Antunes et al. [38] claim that both the hardness and modulus of elasticity increase almost linearly with a filler content. However, bending depends exponentially on filler content and decreases as the amount of filler increases [38].

The device for instrumental evaluation has proven to be very useful in evaluating the quality of roofing sheet coatings. Such a device specifies the force that causes damage to the coating. The device is capable of specifying the critical loads LC1 (first cracks), LC2 (chipping and spatter) and LC3 (coating rupture). The characteristic magnitude of the critical load LC was specified. LC3 is the force which caused critical damage, i.e., significant deformation, cracks and delamination of the coating from the substrate (coating rupture) [39]. The calculated strength of the coating is given in Table 3. Due to difficult interpretation and the need to maintain adequate accuracy of the measurements, the LC3 parameters were evaluated with an optical microscope coupled with a scratch test device. The LC3 force was determined by comparing the microscopic image of the scratch (shows the damage) and the force diagram. Both charts have the same horizontal axis (scratch length). Also used were the plots of acoustic emission (Ae), friction force (Ft), and indenter penetration depth (Pd) as a function of scratch length where characteristic points on the plots were marked.

Additional damage may occur in structures exposed to weather conditions, or structures used while being exposed to corrosive factors because of scratching as a primary reason [35]. It has been demonstrated [40] that damage such as blistering and corrosion occurred only in samples with a scratched coating. In addition, more blistering and corrosion around a scratch occurred on coatings exposed, for a similar amount of time, to varying rather than stable (and therefore more realistic) corrosive conditions [40].

The average values of the LC3 forces are higher for the “white” coating. The variation is, however, not very significant. Moreover, the force values reached for both coatings are not high so the scratch resistance of roof sheet coatings as a qualitative property can be considered as relatively low. Scratch damage itself is a defect in surfaces of roof sheets and can also contribute to the formation of other damage.

The values of the LC3 force are higher for the “white” coating. However, the variation is not very high. Moreover, the force values obtained for both coatings are not high, and it should be considered that the scratch resistance of roofing sheet coatings as a quality characteristic is relatively low. What is worse, the level of this quality feature can also translate into other quality features of sheet coatings. Scratch damage, as such, is a defect in the surface of roofing sheets and may also contribute to the occurence of other defects.

## 4. Conclusions

Organic coatings are common, and many items used on a daily basis, such as furniture, houses, tools, and vehicles, are coated with them for various reasons, including aesthetic, protective, or functional ones [9]. The market of coatings is global and very large [41], so the quality of coatings like resistance to damage due to use is important for a wide range of technical applications. This paper focuses on the comparative evaluation, performed instrumentally, of the quality of roofing sheet coatings in terms of scratch damage resistance. The following final conclusions were formulated from the tests and analysis:

1. The investigated coatings of widely used roofing sheets differ in structure and scratch resistance, so sheet coatings should show an appropriate minimum resistance.
2. The instrumental technique proved to be correct to evaluate scratch resistance in roofing sheet coatings. The scratch resistance of roofing sheet coatings can be evaluated from the LC3 parameter only. However, the values of LC3 for the tested roofing sheet coatings do not differ significantly. Despite the fact that the properties of the coatings determined in the indentation test were significantly different, the instrumental technique should be applied because it gives many more opportunities to evaluate coating properties that result in qualitative features.
3. Given the above, it should be claimed that the research presented in this paper satisfies the needs of technological and operational quality evaluation. The approach of quantitative and qualitative evaluation due to the selected operational exposure, or behavior under concentrated load, is applicative and relatively informative about the mechanical resistance and adhesion to the substrate showed by the investigated coatings, and such findings can provide a basis for the comparison of properties behind coating quality.

## Figures and Tables

**Table 1:** Descriptive statistics of the indentation hardness and surface modulus of the tested materials.

Mechanical Quantity	Statistical Parameter	White Coating	Black Coating
*H_IT_* (O&P) [MPa]	Mean	60.409	14.019
	Std. Dev.	22.7	8.982
	N	5	6
*HV_IT_* (O&P) [Vickers]	Mean	12.029	1.298
	Std. Dev.	4.651	0.832
	N	5	6
*E_IT_* (O&P) [MPa]	Mean	23.336	69.761
	Std. Dev.	8.332	178.840
	N	5	6
E* (O&P) [MPa]	Mean	26.594	535.340
	Std. Dev.	11.291	231.27
	N	5	6

**Table 2:** Findings for the coating surface roughness profile tests.

Roughness Parameter	ZA200—“Black”	S220GD—“White”
*Ra* [μm]	3.074	0.477
*Rt* [μm]	46.256	8.666

**Table 3:** Descriptive statistics of the results of the adhesion tests of the roof sheet coatings.

	ZA200—“Black”	S220GD—“White”
	**LC3 Optic [N]**	
Mean	4.259	3.290
Std. Dev.	0.628	0.542
Min	3.513	2.576
Max	5.716	4.171
N	12.000	11.000
Median	4.099	3.236
	**LC3 F_T_ [N]**	
Mean	3.797	3.000
Std. Dev.	0.667	0.663
Min	2.923	2.274
Max	5.045	4.196
N	9.000	7.000
Median	3.811	2.896
	**LC3 A_E_ [N]**	
Mean	3.587	2.983
Std. Dev.	0.668	0.702
Min	2.793	1.997
Max	4.432	3.987
N	8.000	8.000
Median	3.160	2.866
	**LC3 Pd [N]**	
Mean	3.984	3.291
Std. Dev.	0.774	0.651
Min	2.911	2.501
Max	5.598	4.333
N	12.000	11.000
Median	3.770	3.525

## Data Availability

Data sharing is not applicable to this article.
